# Transcranial sonography window description: a proposal for a rating system

**DOI:** 10.1186/s13089-025-00428-2

**Published:** 2025-07-31

**Authors:** Giada Cucciolini

**Affiliations:** 1 Department of Surgical, Medical, Molecular Pathology and Critical Care Medicine, University of Pisa, Via Savi 10, 56126 Pisa, Italy; 2 Departmental Structure of Neuroanesthesia and Intensive Care, Azienda Ospedaliero-Universitaria Pisana, Via Paradisa 2, 56124 Pisa, Italy

**Dear editor,**

Technological developments in ultrasound devices have allowed an easier visualization of the brain with better image quality, allowing the method to be spread. Transcranial colour-coded Doppler (TCCD) has been applied especially in the neuro-intensive care unit (ICU) and neurology environment [1, 2], even if applications might be extendable to other settings as the general ICU environment [3, 4].

However, description of the transcranial window has been classically reported as qualitative (present or not) with almost 10% of people reported to have not a sufficient transcranial window to explore the brain, especially if they are elderly females [5]. In my experience, this description of the TCCD window is not always representative of the real capacity of the ultrasound (US) beam penetration, that might have different nuances, even when the settings have been optimised.

Different characteristics of the bidimensional images and Doppler insonation can in fact be described. In particular, structures that are more difficult to insonate become progressively clearer as the quality of the acoustic window improves. Regarding the bidimensional imaging the most easily identifiable structures are the contralateral temporal bone and the sphenoid wing. When the window is better, the mesencephalon is generally the next easier identifiable structure followed by the other parenchymal structures (e.g. thalami, ventricular structures, pineal gland, tentorium). Regarding the vessels insonation, even if the bidimensional image is poor, the middle cerebral artery or the posterior cerebral artery might be visible. Conversely, sometimes the bidimensional image might be good or adequate but vessels might be not visible.

To fill the gap between the current qualitative assessments and what we observe in our everyday clinical practice, I propose here a rating system of the transcranial US window that is composed by a number for the bidimensional image quality and a letter for the visible vessels (colour function mode). The combination of numbers and letters composes the final rating as described in Table 1. The distinction of the image and the vessels rating is justified by the different clinical questions that could be answered by these two different aspects of the exam. Two practical examples will be given where a different rating for the TCCD window might justify different clinical decisions. Examples of images and their rating is provided in Table 2.

**Table 1:** Ratings for the transcranial color-coded doppler window

Bidimensional image description	Rating	Colour-Doppler function description	Rating
No structures visible, no US beam penetration	4	No vessels visible	D
Contralateral temporal bone and sphenoid wing visible—bony structures	3	One main vessel only visible (generally MCA or PCA)—one side	C
All the above plus some (but not all) parenchymal structures visible (for example: mesencephalon or third ventricle only visible)	2	More than one main vessel visible, not all explorable	B
All the main parenchymal structures visible (i.e. mesencephalon, pineal gland, thalami, ventricular structures, tentorium cerebelli)	1	All the Willis’ polygon vessels visible (at least MCA, ACA, PCA). Syphon and ICA visible	A

The rating is intended with the best device settings applied (e.g. probe frequency, depth, focus, pulse repetition frequency, scale, filtering).

*ACA* anterior cerebral artery, *ICA* internal carotid artery, *MCA* middle cerebral artery, *PCA* posterior cerebral artery, *US* ultrasound

**Table 2:** Images examples of the rating system

Bidimensional image description	Rating	Colour-Doppler function description	Rating
No US beam penetration. A TCCD window is not identifiable	4	No vessels visible. Mesencephalon is indicated by the dashed line. 4 points star: sphenoid wing	D
A patient in which the bony structures are the only visible structures. The dashed line indicates the sphenoid wing	3	In this image the only vessel identifiable is the MCA. Mesencephalon is indicated by the dashed line	C
Mesencephalon is the only parenchymatous structure clearly identifiable in this patient (indicated by the dashed line)	2	The vessels identifiable are the middle and posterior cerebral artery. Anterior cerebral artery is not visible even changing the probe positioning. Dashed line: mesencephalon. M1: middle cerebral artery first tract. M2: middle cerebral artery tract 2. P1: posterior cerebral artery first tract. P2: posterior cerebral artery tract 2	B
TCCD window where all the principal parenchymal structures are identifiable. In this case we can see the third ventricle (4-points star) and the pineal gland (arrow)	1	In this image all the Willis polygon vessels are visible. ACA: anterior cerebral artery. cACA: contralateral anterior cerebral artery. M1: middle cerebral artery first tract. M2: middle cerebral artery tract 2. cM1: contralateral middle cerebral artery first tract. PCoA: posterior communicating artery. PCA: posterior cerebral artery	A

Let’s suppose to have a patient in whom brain death is suspected based on clinical criteria. This patient has been sedated until a few hours ago and therefore a test is required to demonstrate the absence of flow in the three main vessels of the intracranial circulation. In this case, if the patient has an excellent transcranial window with a rating A for the vessels at a previous exam, absent or reverberant flow in all the the principal brain vessels could be demonstrated and investigations to determine the cerebral death could proceed. Conversely, if only some of the vessels are visible (rate B), an angiographic study or a 24-h sedation hold is required (according to our local regulations), which would prolong the ICU stay or require patient mobilization, incurring in additional costs and workload.

In people with a good accessibility of TCCD window for parenchymatous structures the exam reliability might be high for detection of the third ventricle enlargement and the calculation of the midline shift. It has in fact been proved that TCCD has a good sensibility and specificity for midline shift detection, and in people with a good view of the intracranial structures TCCD might avoid unnecessary CT scans [6]. However, the sensitivity and specificity of the exam might drop down if the patient has a low US beam penetration and thus a CT scan would be essential for the diagnosis or rule out of hydrocephalus or midline shift when these conditions are suspected.

Regarding research objectives this TCCD window rating might have an impact as well. For example, in patients with previous aneurysmal subarachnoid haemorrhage TCCD examination for vasospasm detection has been reported to have a high positive predictive value only for the middle cerebral artery [7]. Even if the TCD accuracy for vasospasm has been extensively explored, studies on TCCD accuracy are not so numerous and evaluation of the sensibility and specificity could be re-evaluated in relation to the rating of the transcranial window for the vessel’s examination. 

However, the clinical feasibility and research usefulness of this rating system application must be tested in practice and future pilot studies are thus awaited. Anyway, a description of the transtemporal window quality, expressed as a rating, would standardise the TCCD window description helping in understanding the strengths and limitations of the exam. This would allow a better interpretation of the results explaining a low reproducibility of the exam (low inter-rater reliability) or strengthening its results. In addition, it could open different research perspectives regarding the diagnostic accuracy of TCCD for various conditions. For these reasons, the rating of the TCCD window might be considered as an important parameter to include into the report of the exam.

## Supplementary Information

Supplementary Material 1.

## Data Availability

Data sharing is not applicable to this article as no new data were created or analyzed in this study.
