# Supplementary material for: Transcranial sonography window description: a proposal for a rating system
Source: Ultrasound J. 2025 Jul 31;17:36. doi: 10.1186/s13089-025-00428-2 (PMC12314177; doi:10.1186/s13089-025-00428-2)

Examples of transtemporal TCCD window rating

# Case 1

A case of a patient with a brainstem haemorrhage, on the second day from admission. Contralateral bone, third ventricle, lateral ventricles and mesencephalon were visible, with a distortion of the mesencephalon due to haemorrhage. All the vessels in the Willis polygon were visible. The rating here is 1A.


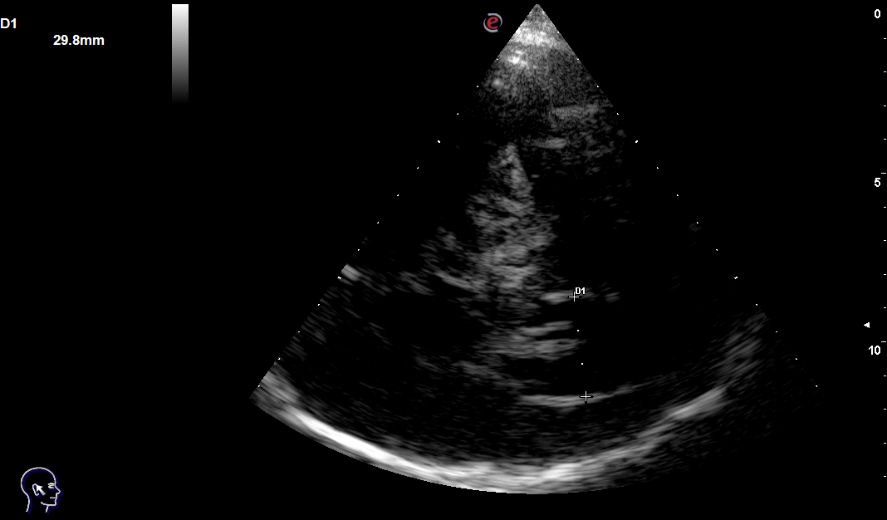

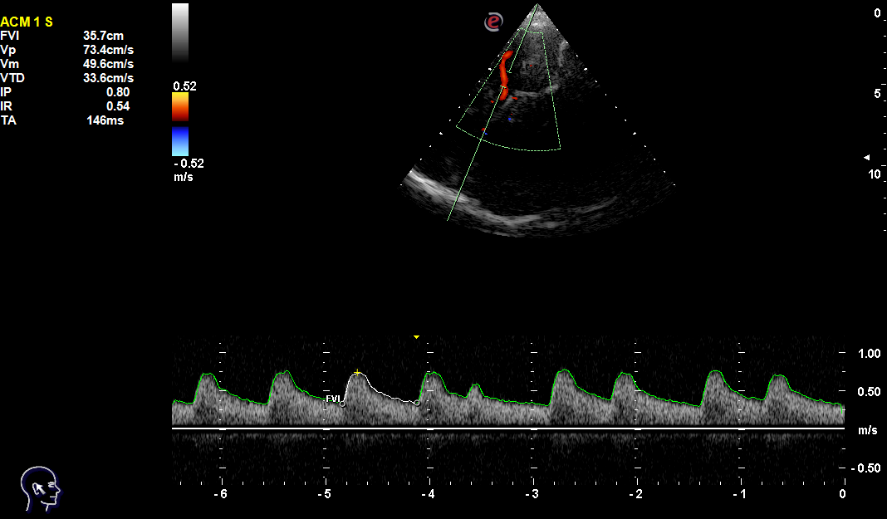

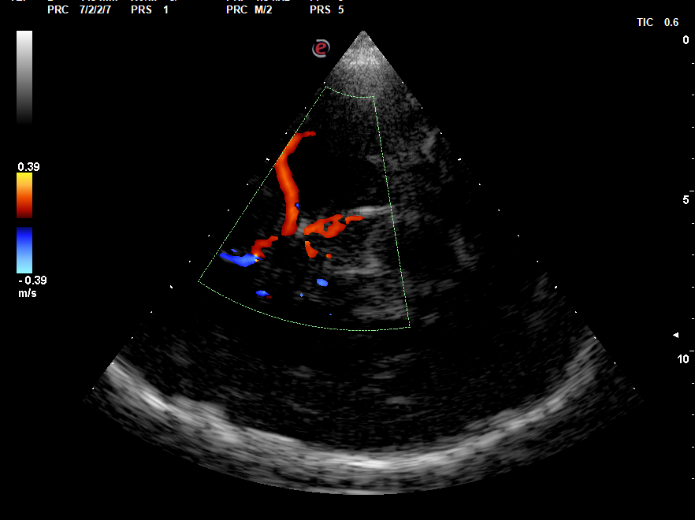


# Case 2

This case is a 78 year old female patient with a spontaneous cerebral hemorrhage that unfortunately died. At the moment of examination the patient had a Glasgow coma scale of 3, with no brainstem reflexes. A trial for a TCCD was performed but no US window was found (only the contralateral temporal bone was visible). To confirm the intracranial hypertension other point of care approaches were used, like the transorbital insonation of the syphon and the measurement of the optic nerve sheet diameter. The rate for the transtemporal window here was 3D.


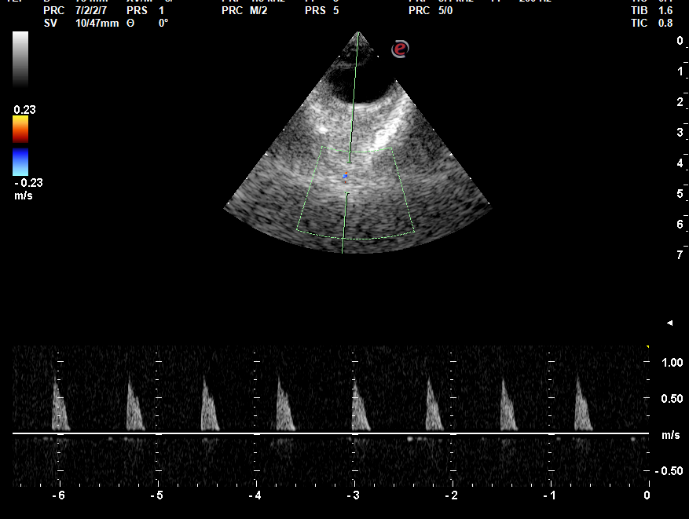

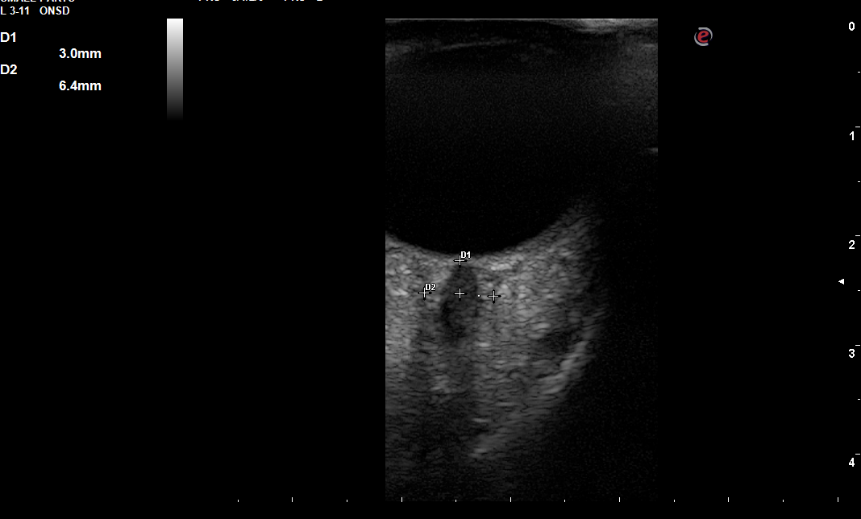

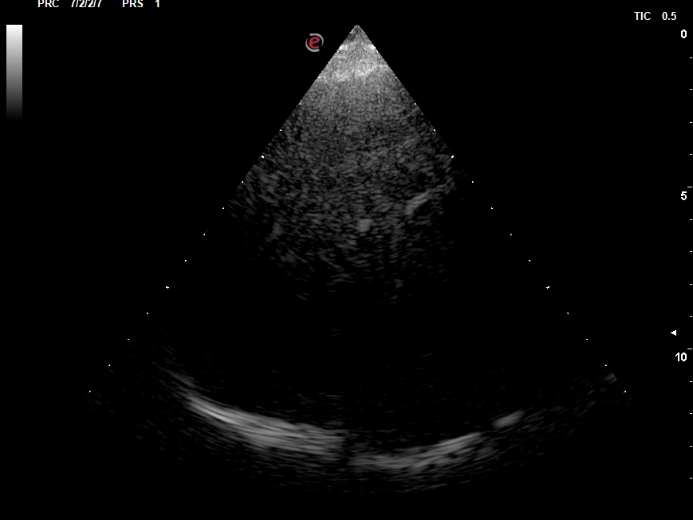


# Case 3

This is a case of a 53 year old male with an ischemic stroke due to an occlusion of the basilar artery, that underwent thrombectomy and developed cerebellar edema. A TCCD evaluation was performed to evaluate the flow of the basilar artery and the other vessels (in order to exclude hydrocephalus). The transcranial window allowed visualization of the mesencephalon (not clearly visible), and the middle cerebral artery. The other vessels of the Willis polygon and the third ventricle were not visible. The basilar artery was insonated via the transforaminal window. In this case the transtemporal window can be rated as a 2C.
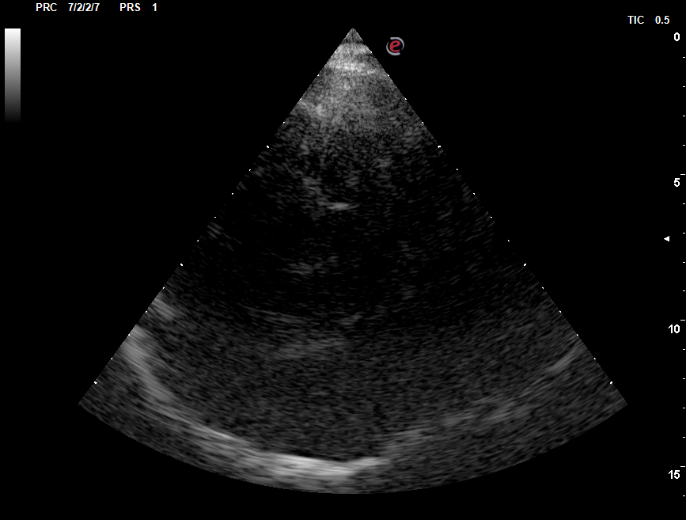


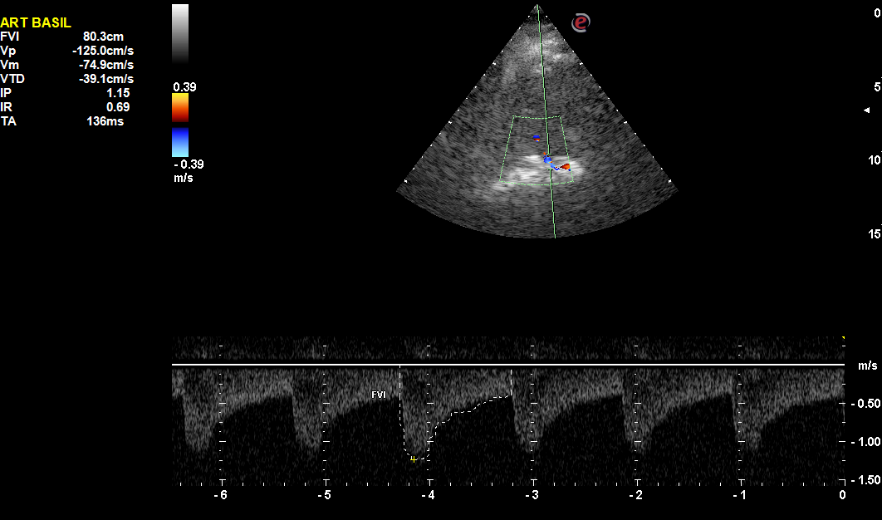

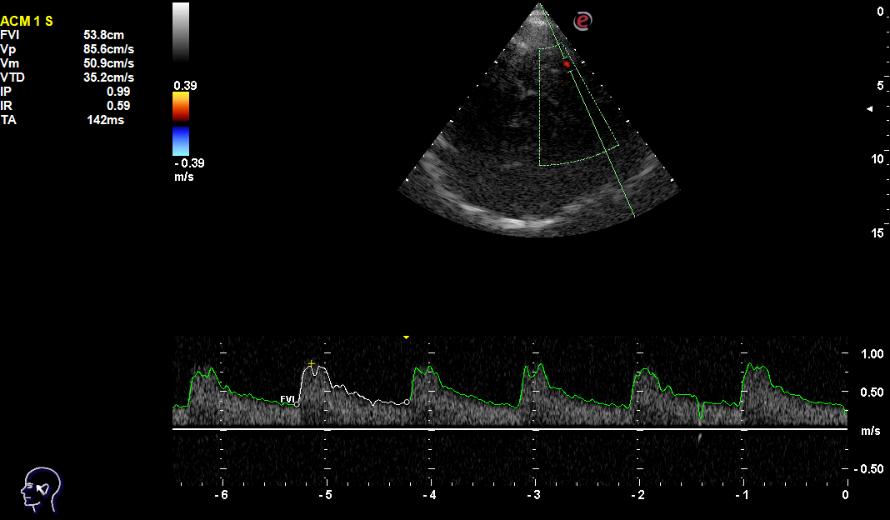

Supplement: Supplementary file 1 — Supplementary Material 1. [file 13089_2025_428_MOESM1_ESM.docx]
